# Identification of *Anncaliia algerae* in Ascites in an Immunosuppressed Patient, China

**DOI:** 10.1093/ofid/ofae393

**Published:** 2024-07-12

**Authors:** Zanzan Wang, Dan Li, Lingling Lu, Zhijuan Xu, Guifang Ouyang, Yongcheng Sun

**Affiliations:** Department of Hematology, The First Affiliated Hospital of Ningbo University, Ningbo, China; Key Laboratory of Digital Technology in Medical Diagnostics of Zhejiang Province, Dian Diagnostics Group Co, Ltd, Hangzhou, China; Hangzhou DA Medical Laboratory, Hangzhou, China; Key Laboratory of Digital Technology in Medical Diagnostics of Zhejiang Province, Dian Diagnostics Group Co, Ltd, Hangzhou, China; Hangzhou DA Medical Laboratory, Hangzhou, China; Department of Hematology, The First Affiliated Hospital of Ningbo University, Ningbo, China; Department of Hematology, The First Affiliated Hospital of Ningbo University, Ningbo, China; Department of Hematology, The First Affiliated Hospital of Ningbo University, Ningbo, China

**Keywords:** albendazole, *Anncaliia algerae*, gastrointestinal involvement, immunosuppressed, mNGS

## Abstract

*Anncaliia algerae*, a microsporidium, has risen to prominence as an opportunistic pathogen, particularly afflicting individuals who are immunocompromised with conditions such as rheumatoid arthritis, organ transplantation, and hematologic malignancy. Surprisingly, despite its recognized impact, the identification of *A algerae* in ascitic fluid has not been documented. As such, we pinpointed *A algerae* as the probable instigator of ascitic accumulation in a patient with a history of acute myeloid leukemia and extended periods of immunosuppressive therapy. For this patient, there were no signs of *A algerae*–related infections (eg, myositis), vocal cord involvement, or disseminated infection. The presence of *A algerae* was finally identified by next-generation metagenomic sequencing analysis of the ascitic fluid. Clinical presentation was characterized by elevated C-reactive protein levels (110.7 mg/L), diminished platelet count (48 × 10^9^/L), abdominal distension secondary to ascitic fluid accumulation, and lower limb pain, and it showed marked improvement following a 4-day regimen of sulfamethoxazole/trimethoprim and albendazole. Despite this promising response, the patient succumbed to aspiration of vomitus. This case underscores the importance of considering rarer organisms, such as *A algerae* infection, in patients who are immunocompromised and present with unexplained ascites accumulation. It highlights the potential effectiveness of sulfamethoxazole/trimethoprim and albendazole in managing such cases. Further research is warranted to elucidate optimal management strategies and improve outcomes in similar clinical scenarios.

Microsporidia, obligate intracellular parasites closely related to fungi, possess the capability to infect nearly all animal taxa, making them the fourth-most prevalent cause of infection in humans [1]. *Encephalitozoon* and *Enterocytozoon* represent 2 genera commonly implicated in human pathogenicity [2]. *Anncaliia algerae*, a microsporidian species predominantly observed in insects, was initially documented in 2004 in a patient with fatal myositis [3]. Since then, it has emerged as an opportunistic pathogen affecting individuals who are immunocompromised with conditions such as rheumatoid arthritis, organ transplantation, and hematologic malignancies [1, 4–7]. While myositis is the prototypical manifestation of *A algerae* infection, previous cases have documented skin and vocal cord involvement, as well as disseminated infections [1, 4, 8]. To date, the identification of *A algerae* in ascites fluid has not been reported. Among documented cases, successful management was reported in only 2 cases [9, 10]. Given the inherent risk of fatality, precise diagnosis and timely intervention are imperative.

We report a case of *A algerae* microsporidiosis involving ascitic fluid in a female who was immunocompromised, providing a detailed account of the diagnostic timeline and the treatment modalities employed.

## CASE REPORT

**Figure 1.**
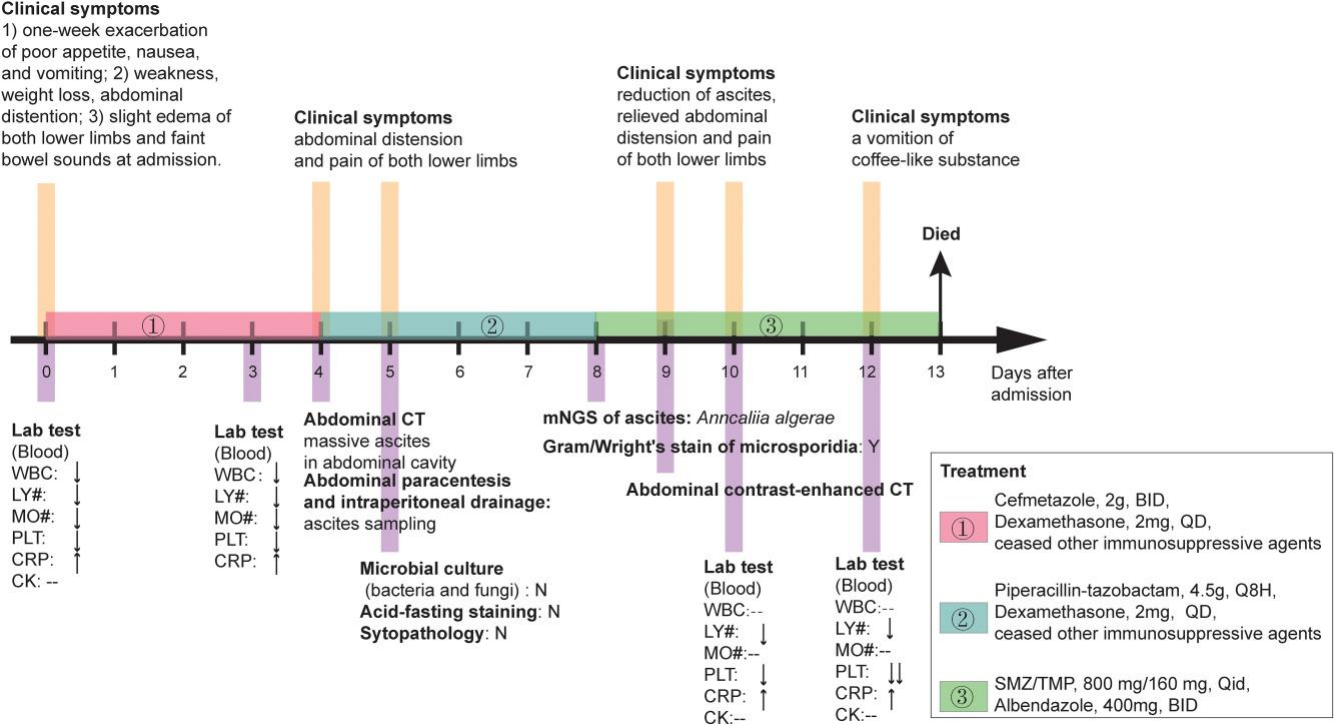
Clinical course of a 63-year-old woman with *Anncaliia algerae* microsporidian infection, China. Abbreviations: WBC, white blood cell; LY, lymphocyte; MO, monocyte count; PLT, platelet; CRP, C-reactive protein; CK, creatine kinase; N, negative identification of *Anncaliia algerae*; Y, positive identification of *Anncaliia algerae*. ↑，more than normalization; ↓, less than normalization. #, absolute count; --, normal level.

A 63-year-old woman residing in China sought medical attention at a hospital, reporting a 1-month history of discomfort marked by poor appetite, nausea, and vomiting. The escalation of symptoms for 1 week prompted her admission to the hospital (Figure 1). Upon clinical evaluation, she was afebrile and free of hoarseness, sore throat, or cough, alongside weight loss, weakness, and abdominal distension. The patient's medical history revealed a diagnosis of myelodysplastic syndrome 10 years prior, which progressed to acute myeloid leukemia 3 years ago. Following allogeneic hematopoietic stem cell transplantation (allo-HSCT) combined with long-term immunosuppressive therapy, her bone marrow remained in complete remission. In the preceding year, she had been receiving ruxolitinib (5 mg orally, twice daily), prednisone (10–20 mg orally, daily), and methotrexate (10 mg orally, weekly) due to chronic skin graft-vs-host disease. Long-term use of prednisone resulted in femoral head osteonecrosis, resulting in persistent pain in both lower limbs over the past year. Additionally, the patient had a history of hepatitis B over a decade, for which she was treated with entecavir.

Physical examination of the patient revealed slight edema of both lower limbs, faint bowel sounds, and abdominal tenderness but no muscle tenderness in the limb. The respiratory sounds of both lungs were clear without dry or wet rales, and cardiac examination revealed no murmur. Laboratory investigations showed a moderate elevation in C-reactive protein levels to 122.99 mg/L, a decrease in platelet count to 105 × 10^9^/L, and lymphocyte and monocyte counts <1.0 × 10^9^/L. The white blood cell count was 2.1 × 10^9^/L with 86.2% neutrophils, 10.5% eosinophils, and 1.4% basophils. In addition, liver and kidney function tests were done. The value of alanine aminotransferase was 121 U/L, aspartate aminotransferase was 144 U/L, serum creatinine was 36 μmol/L, and blood urea nitrogen was 5.8 mmol/L. In addition, creatine kinase levels were monitored every other day, with no elevations exceeding 50% of the upper limit of the reference range observed throughout the hospitalization. Echocardiography was performed on the fourth hospital day and showed no abnormal findings. The cardiac systolic and diastolic functions were normal. The B-scan ultrasound of the liver showed normal size and morphology with uniform density. Moreover, the B-scan ultrasound did not show high portal pressure. Initial treatment involved administering 2 mg of dexamethasone daily while discontinuing other immunosuppressive agents. Intravenous immunoglobulin and broad-spectrum antimicrobial therapy were also initiated. Given the clinical presentation, intestinal obstruction was considered a primary concern, providing timely and continuous gastrointestinal decompression.

**Figure 2.**
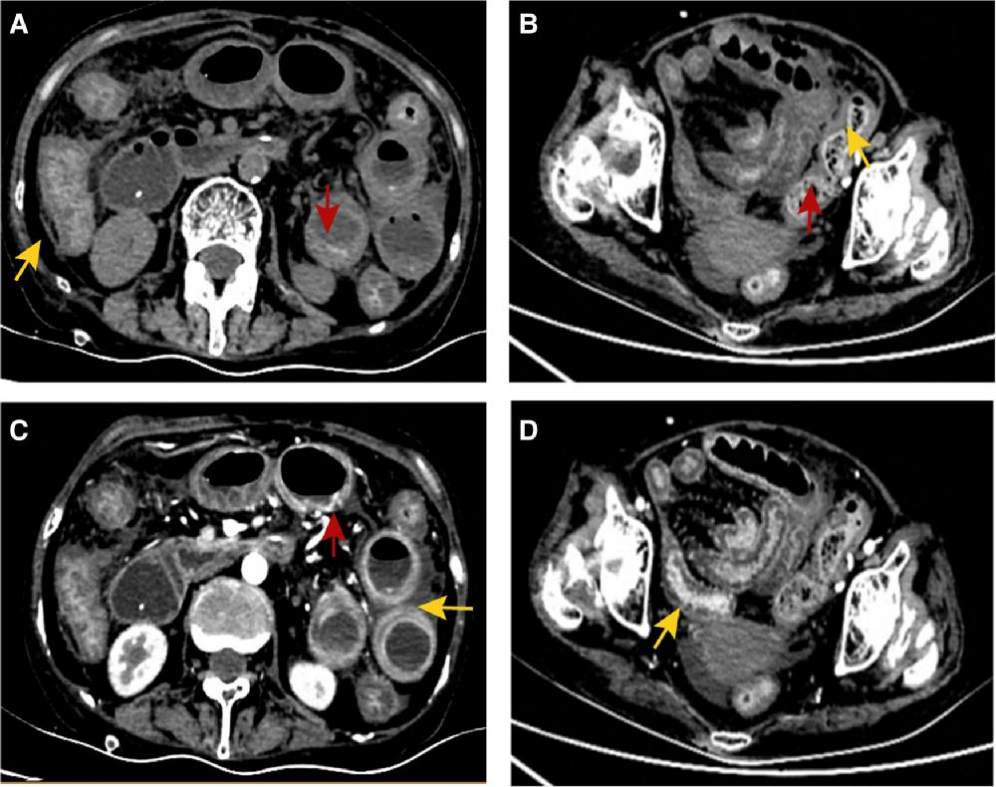
Representative image of abdominal computed tomography: *A* and *B*, scan of abdomen; *C* and *D*, contrast-enhanced scan of abdomen. A thickened bowel wall and the edema of mucosa are indicated (yellow arrows). Linear and annular high-density shadow on mucosal surface indicates mucosal hyperemia (red arrows). Meanwhile, blurred fat gaps around the bowel wall suggest effusion in the abdominal cavity.

Despite interventions, the patient's condition continued to deteriorate, with complaints of worsening abdominal distension and progressive aggravation. Concurrently, C-reactive protein levels continued to escalate (302.85 mg/L) while platelet counts sharply declined (19 × 10^9^/L), suggesting an aggravated infection potentially leading to secondary thrombocytopenia characterized by a rapid decline in platelet count. Subsequent computed tomography scans of the abdomen revealed no abnormality in the liver, gall bladder, kidney, pancreas, uterus, and bladder. However, notable findings included thickening of the intestinal wall, blurred fat spaces around the intestinal wall, mucosal edema, and massive ascites in the abdominal cavity (Figure 2). Ultrasound-guided puncture and drainage of ascites were performed, and a pig-tail catheter was inserted into the peritoneal cavity to collect light yellow turbid ascites daily, yielding approximately 600 to 800 mL over a 4-day period. Subsequent ascitic biochemical analyses revealed a positive Rivalta test result, with total protein levels at 28.4 g/L, albumin at 20.9 g/L, globulin at 7.5 g/L, and lactate dehydrogenase at 856 U/L. Additionally, laboratory findings indicated 476 leukocytes/µL in the ascitic fluid, comprising 51% lymphocytes, 23% neutrophils, 1% eosinophils, and 25% mesothelial cells. Notably, acid-fast staining, microbial culture (bacteria and fungi), and cytopathology of the ascitic fluid all yielded negative results. Stool microscopy was done, but microsporidia were not detected.

**Figure 3.**
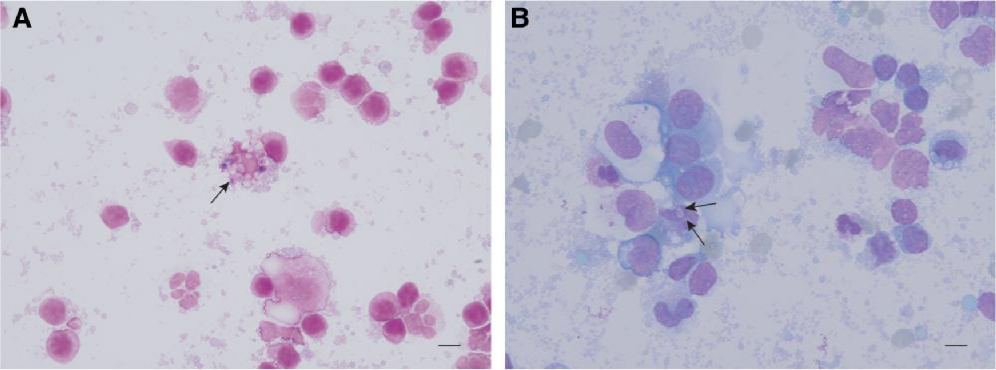
Light microscopy of ascites from a 63-year-old woman infected by *Anncaliia algerae*: *A*, gram stain; *B*, Wright stain. Black arrows indicate the cluster of ovoid organisms within ascites.

On the eighth day of admission, microscopic examination of the ascites with special stains (gram and Wright) revealed a cluster of 2- to 3-μm ovoid spores. However, microscopy alone was insufficient to identify the microsporidian genus and species (Figure 3). Metagenomic next-generation sequencing (mNGS) was then performed to elucidate potential underlying causes for the ascites. By combining the clinical characteristics with the mNGS results, the pathogen was further identified as *A algerae* with a relative abundance of 20.5% (Table 1, Supplementary Material 1). Subsequently, a treatment regimen consisting of sulfamethoxazole/trimethoprim (SMZ/TMP; 800 mg/160 mg, 4×/d) and albendazole (400 mg, 2×/d) was established on the basis of previous successful reports [9, 10].

**Table 1.** Identification of *Anncaliia* Genus From Ascites Based on Metagenomic Next-Generation Sequencing

Genus			Species		
Name	Relative Abundance	Sequences	Name	Confidence Level	Sequences
*Anncaliia*	20.5%	6	*Anncaliia algerae*	99%	6
*…*	…	…	Torque teno virus	99%	10

Pathogens are classified according to bacteria, fungi, viruses, and parasites, and relative abundance is the relative proportion of genomes in the corresponding classification of the pathogen. Sequences refer to high-throughput sequencing sequences that are uniquely aligned to genus- or species-specific sequences. Confidence level is defined as the reliability of the identified pathogen in the sample based on sequence alignment. For clinically pathogenic bacteria, fungi, and virus, if the reads ≥3 and ≥ that in the negative controls of same batch was determined as positive.

After 4 days of treatment, notable symptomatic improvement was observed during clinical rounds, as evidenced by a reduction in ascites volume. Furthermore, the restoration of C-reactive protein levels (110.7 mg/L) and platelet counts (48 × 10^9^/L) suggested the efficacy of SMZ/TMP and albendazole against *A algerae* infection. Unfortunately, the patient experienced vomiting of a coffee-like substance, likely due to the ingestion of semisolid food, followed by aspiration, leading to significantly low oxygen saturation. Subsequently, her family elected to discontinue life-sustaining therapy upon her loss of consciousness, and she died the first day thereafter.

## DISCUSSION

Infections attributed to *A algerae* have been documented across a spectrum of cases, predominantly manifesting in individuals who are immunocompromised (Table 2), implicating immunosuppression as a predisposing factor for *A algerae* infection. The patient under discussion exhibited severe immunosuppression owing to the management of allo-HSCT and prolonged immunosuppressive therapy. Nonetheless, speculations regarding potential routes of transmission include ingestion, inhalation, and direct inoculation, but the precise mode of *A algerae* transmission remains indeterminate [11, 12]. Notably, the patient resided in a humid rural area in the southern region, where exposure to water sources potentially contaminated with *A algerae* spores was conceivable. The presence of abdominal abnormalities further suggested a plausible gastrointestinal route of entry.

**Table 2.** Case Literature of *Anncaliia algerae* Microsporidiosis

Author	Country	Disease History	Clinical Diagnosis	Detected Samples	Positive Detection Methods	Outcome
Anderson 2019 [4]	…	Pancreas and renal allograft recipient	Disseminated *A algerae* infection	Lower extremity, finger, and tongue biopsies	PCR	Died
Field 2012 [5]	…	Bilateral lung transplant	Myositis	Tongue and deltoid muscle	PCR	Died
Cali 2010 [1]	Newark, NJ	Chronic lymphocytic leukemia	Human vocal cord infection	Vocal cord nodules	PCR	Died
Coyle 2004 [3]	Bronx, NY	Rheumatoid arthritis	Myositis	Muscle	PCR	Died
Boileau 2016 [9]	Canada	Chronic lymphocytic leukemia	Myositis	Muscle	PCR	Recovery
Sutrave 2018 [10]	Australia	Hemopoietic stem cell transplantation	Myositis	Vastus lateralis	PCR	Recovery
Ziad 2021 [7]	New Zealand	Psoriatic arthritis	Myositis	Vastus lateralis	PCR	Died
Liu 2022 [6]	China	Kidney transplant	Myositis	Muscle	mNGS	Died

Abbreviations: mNGS, metagenomic next-generation sequencing; PCR, polymerase chain reaction.

To the best of our knowledge, this is the first description of *A algerae* microsporidia within ascites (Table 2). It is worth mentioning that the patient had a chronic hepatitis B virus infection, necessitating further investigation into the cause of the ascites. Following allo-HSCT, circulating hepatitis B surface antigen and hepatitis B virus DNA were monitored every 6 months at outpatient clinics. Serum hepatitis B surface antigen expression was low, and hepatitis B virus DNA remained undetectable. Aspartate aminotransferase and alanine aminotransferase levels were normal or slightly elevated during monthly reviews. Additionally, neither ultrasound nor computed tomography scans showed evidence of portal hypertension. Therefore, the ascites might be related to an acute infection rather than portal hypertension due to chronic liver disease. Characteristically, myalgias, fever, and fatigue represent typical manifestations of *A algerae* infection, with concurrent reports of neurologic complications [5, 10]. Notably, the patient under examination herein maintained normal levels of creatinine kinase throughout hospitalization and exhibited no muscle tenderness, leading to the inference that skeletal muscle involvement was absent. Nevertheless, the absence of lower limb electromyography or magnetic resonance imaging investigations presented a limitation in substantiating this conclusion. The patient endured symptoms of nausea, vomiting, abdominal distension, and ascitic accumulation attributed to microsporidia, thereby elucidating updated clinical presentations of *A algerae* infection. It is imperative for clinicians, pathologists, and microbiologists to exercise vigilance toward microsporidiosis during the diagnostic process, particularly in individuals who are immunocompromised and present with ascitic accumulation.

Due to the inherent risk of fatality associated with *A algerae* infection, early diagnosis and expeditious intervention are imperative. Regarding the diagnosis of microsporidiosis, the conventional approach involves culturing isolates, followed by identification via light microscopy or transmission electron microscopy, supplemented with specific staining in conjunction with polymerase chain reaction diagnostics (Table 2) [4, 12, 13]. However, sole reliance on light microscopy for morphologic diagnosis is difficult to accurately distinguish the genus and species of microsporidia [4, 5]. Although polymerase chain reaction increases the diagnostic reliability, it is difficult to directly identify uncommon pathogens [14, 15]. mNGS presents as a culture-free, unbiased, and time-efficient method for the identification of a broad spectrum of pathogens, showcasing an adjunctive tool in clinical diagnosis, particularly for infrequent pathogens such as *A algerae* infection [16,17–18].

Successful management of *A algerae* infection has been documented in 2 cases. However, the optimal therapy remains undetermined. In clinical practice, SMZ/TMP is considered a safe, efficient, and low-cost therapy that exhibits broad-spectrum antibacterial activity by suppressing bacterial folic acid synthesis [19–21]. Albendazole, an antitubulin polymerization agent used to combat parasitic worms, has shown efficacy in treating microsporidian infections [22]. Mechanically, the ß-tubulin amino acid sequence from *A algerae* contains 5 conserved amino acid sequences linked to benzimidazole sensitivity, leading to the inhibition of new spore production and release, as well as abnormalities in the developing parasite [22]. Notably, in cases of *A algerae* infection, patient recovery ensues following fumagillin supplementation, whereas albendazole alone proves mitigating but not curative [9]. In this case, the efficacy of the combination of albendazole and SMZ/TMP against *A algerae* infection was not directly assessed, but the alleviation of clinical symptoms suggested its utility in treating *A algerae* infection. The patient's demise was presumed to be attributed to aspiration of vomit.

This is the first case of *A algerae* found in ascitic fluid in a patient undergoing immunosuppressive therapy. However, our study was subject to certain limitations that warrant consideration for further investigation. Microsporidiosis was initially misdiagnosed as intestinal obstruction because of the lack of etiology and clinical experience of *A algerae* infection, which might delay the treatment. While the clinical features of *A algerae* infection have been obtained from a series of cases, the absence of comprehensive epidemiologic data underscores a need for continued accumulation of cases associated with *A algerae* infection. Subsequent accumulation of such data would facilitate a more comprehensive analysis, enabling the exploration of potential variations in the presentation of *A algerae* infection across diverse populations. In addition, while the utility of albendazole and SMZ/TMP in treating *A algerae* infection could be inferred from clinical characteristics, real-time monitoring of pathogen clearance would provide more convincing evidence. Ultimately, this approach would enhance the precise identification and management of this disease in the future.

## CONCLUSION

In summary, the case study contributed to a more comprehensive comprehension of the clinical manifestations associated with *A algerae* infection. It underscored the necessity for a heightened index of suspicion, particularly regarding ascitic fluid accumulation in patients who are immunocompromised, which might signify an underlying but potentially fatal microsporidiosis. Moreover, the application of mNGS has emerged as a promising adjunctive tool, especially for clinical uncommon or emerging pathogens.

Furthermore, targeted therapy combining albendazole and SMZ/TMP demonstrated utility in mitigating *A algerae* infection, albeit with varying degrees of success. However, a thorough investigation into the epidemiologic landscape and underlying pathogenic mechanisms of *A algerae* infection is imperative. Such endeavors are paramount for the identification of optimal therapeutic modalities and the development of more effective treatment strategies.

## Supplementary Material

ofae393_Supplementary_Data

## Supplementary Data

Supplementary materials are available at *Open Forum Infectious Diseases* online. Consisting of data provided by the authors to benefit the reader, the posted materials are not copyedited and are the sole responsibility of the authors, so questions or comments should be addressed to the corresponding author.

## Notes

***Author contributions.*** L. L. and Y. S. conceived the case. D. L., Z. W., Z. X., and Y. S. were responsible for the data collection. D. L. and Z. W. combed the case. L. L. conducted the mNGS detection of the sample. D. L. and Z. W. prepared and revised the manuscript. Z. X., G. O., and Y. S. commented on the manuscript. All authors approved the final version of the manuscript for publication.

***Data availability.*** The data of this study can be obtained from the corresponding authors upon reasonable request.

***Patient consent statement.*** The study was conducted in accordance with the Declaration of Helsinki and approved by the Ethics Committee of the First Affiliated Hospital of Ningbo University. The requirement for informed consent was waived because it was a retrospective study and the patient information has been anonymized.

***Financial support*.** This work was supported by the Ningbo Science and Technology Plan Project (No. 2022S032).
