# Supplementary material for: Identification of Anncaliia algerae in Ascites in an Immunosuppressed Patient, China
Source: Open Forum Infect Dis. 2024 Jul 12;11(8):ofae393. doi: 10.1093/ofid/ofae393 (PMC11295253; doi:10.1093/ofid/ofae393)
Supplement: ofae393_Supplementary_Data [file ofae393_supplementary_data.docx]

**Supplementary Material 1**

**DNA Extraction, Library Preparation, and Sequencing**

Ascites samples were collected based on standard clinical procedures. DNA was extracted from the ascites samples following the manufacturer’s instructions (Genskey Co., Ltd., Beijing, China). Effective host DNA removal was achieved using magnetic beads to reduce high contamination from the host genome. DNA library construction involved DNA enzyme digestion, end-repair, barcode ligation, and PCR amplification using the NEBNext Ultra II DNA Library Prep Kit (New England Biolabs Inc.), according to the manufacturer’s instructions.[1] The quality of the DNA library was assessed using the Qubit dsDNA High-Sensitivity (HS) Assay Kit (Thermo Fisher Scientific, USA) before sequencing. The MGISEQ-200RS high-throughput sequencing reagent kit (FCL SE50) was used. According to the manufacturer’s instructions, the library pool was sequenced using the MGISEQ-200RS sequencing platform (MGI, Shenzhen, China) to generate approximately 20 million reads.[2] For each run, human nucleic acid as a negative control was detected in parallel using the same protocol to calculate background levels and monitor sample-to-sample contamination.[1]

**[Bioinformatics analysis]**

Subsequently, fastp software was used to remove low-quality reads, adaptor contamination, and duplicate reads, as well as those shorter than 50 bp. Human sequence data were identified and excluded by mapping to a human reference genome (Genome Reference Consortium Human Build 38, GRCh38) using Burrows-Wheeler Aligner (BWA).[3] After host DNA removal, the filtered data of each sample were standardized to 20 million reads. The filtered data were then aligned to an in-house genome database (Dian Diagnostics Pathogenic Microorganism Genome Database) using BWA software. Annotation and statistics on the sequence reads were subsequently performed. For species-specific sequence homogenization, microbiota prevalence was determined according to mNGS criteria.

**[Criteria of mNGS positive results]**

For clinical core pathogens and bacteria that are difficult to detect, such as *Firmicutes* and intracellular bacteria, the presence of one read was considered positive. For other clinically relevant bacteria, fungi, and viruses, the presence of three or more reads, and reads exceeding those in the negative controls of the same batch, was considered positive. For bacteria, fungi, and viruses that have not been clinically reported or isolated, the presence of 20 or more reads and reads exceeding those in the negative controls of the same batch was considered positive. For parasites, 20 or more reads, and for special pathogens, such as Entamoeba histolytica, one read was considered positive.[4]

**Reference**

[1] Li Y, Li GY, Han DX, Feng YW, Liang XM, Ren D, et al. Study on the clinical indications for plasma as an alternative to the bronchoalveolar lavage fluid metagenomic next-generation sequencing test to detect consistent pathogens for septic patients in intensive care units. Journal of Infection 2022;85(4):442-5. doi:10.1016/j.jinf.2022.07.016.

[2] Miller S, Naccache SN, Samayoa E, Messacar K, Arevalo S, Federman S, et al. Laboratory validation of a clinical metagenomic sequencing assay for pathogen detection in cerebrospinal fluid. Genome Research 2019;29(5):831-42. doi:10.1101/gr.238170.118.

[3] Li H, Durbin R. Fast and accurate short read alignment with Burrows-Wheeler transform. Bioinformatics 2009;25(14):1754-60. doi:10.1093/bioinformatics/btp324.

[4] Luo W, He YB, Xu JH, Zhang SH, Li CX, Lv JF, et al. Comparison of Third-Generation Sequencing Technology and Traditional Microbiological Detection in Pathogen Diagnosis of Lower Respiratory Tract Infection. Discovery Medicine 2023;35(176):332-42. doi:10.24976/Discov.Med.202335176.34.
